# Role of Ophthalmic Nurses in Prevention of Ophthalmic Diseases

**Published:** 2013

**Authors:** Mohammad Bagher Hadavand, Fatemeh Heidary, Roghayeh Heidary, Reza Gharebaghi

**Affiliations:** 1 Health Insurance Organization, Tehran, Iran,; 2 Shiraz University of Medical Sciences, Shiraz, Iran,; 3 Alborz University of Medical Sciences, Karaj, Iran

**Keywords:** Ophthalmic Nurse, Prevention, Ophthalmic Diseases, Visual Impairment, Optometry, Health-care Systems

## Abstract

There are numerous ocular problems that could be diagnosed and detected by well-trained ophthalmic nurses. Ophthalmic nurses may significantly contribute in ophthalmology and visual sciences. These trained nurses may assist in decrease the rate of cancelled surgical operations at the date of operation that had been due to lack of attention to health problems in pre-operative assessments. Furthermore, they could perform some medical evaluation of patients that are candidates for surgery, preoperatively. Additionally, their services would be beneficial to accelerate discharging patients, which would result in less drain on financial resources for medical centres. Ophthalmic nurses are also critical elements in health-care systems because they can help to deliver up to date ophthalmic knowledge and contribute to general physicians, academically. Additionally, they may be able to assist patients who suffer from blindness or loss of vision, to find national organizations that provide services and education. They are able to be a great assistance in referring patients that need subspecialty services and subsequently, contribute to saving health-care expenditures by let the patients to receive proper management. These nurses could play a significant role in the process of teaching people, providing proper diagnoses, administration, and management of ocular problems.

## INTRODUCTION

Development in healthcare services and management of patients are progressing rapidly as are new kinds of ophthalmic diseases, which has created a need for many professionals in health-care disciplines to study and become aware of such developments. One aspect of adopting this approach is the role of ophthalmic nurses, who can play a crucial part in the subspecialty area of diagnosis and management. 

 In modern health-care systems, ophthalmic nurses are well recognized in management of medical and emergency conditions and they could spend more time with ophthalmic patients than others. In many simple cases, they will replace the ophthalmologist by virtue of their relationship with patients (1,2). Specialized hospitals, unlike general hospitals, often provide a higher amount of medical and nursing care for their patients. Because of this, along with the increasing growth of new disciplines in ophthalmology and visual sciences as well as inter-disciplinary fields, makes it fortuitous to arrange and plan for more training courses in ophthalmic nursing, along with refreshment courses and continuous education programs. Through collaboration, nurses and physicians will become better able to provide a higher level of medical care and meet their patients’ expectations. Given the specialized knowledge of nurses and their assistance in managing patients, nurses could be great sources of assistance to ophthalmologists and in this way, contribute to the progress of the healthcare services.

## Visual impairment; a worldwide crisis

Visual impairments result from many reasons and by adopting a healthy lifestyle and/or by arranging screening tests, many of those complications and impairments could be prevented. Many diseases could be easily prevented or pre-diagnosed through monitoring particular groups of people and evaluating their risk factors (3). Many common complications and health risks in today’s world such as diabetes and hypertension could be prevented or controlled through assessing and checking the risk factors prior to the progression of the disease. Those living in developing countries are especially susceptible to many ocular diseases such as diabetic retinopathy, glaucoma, macular degeneration, cataracts and trauma. On a global level, visual impairment is considered as one of the health risks. According to the World Health Organization (WHO), there are around 314 million people in the world who, to some extent suffer from vision impairment and 45 million who do not have accessibility for eye care. However, around 80% of these visual impairments could be easily treated or prevented by taking some pre-emptive measures. Nonetheless, due to lack of ophthalmological services, millions of people risk losing their vision (3,4).

 In order to ensure that nurses could perform their duties in an effective manner, they are likely to require special training. At the very least, they need to understand the basic functions of the eye and learn rudimentary information and knowledge about ocular diseases and complications. Nurses could support the process of categorizing eye pathologies in terms used in emergency or ordinary medical situations and perform triage by identifying benign and malignant conditions. In addition, nurses should receive training on the psychological impacts of vision loss or impairment in order to be better able to assist patients and communicate effectively with them. They should be able to treat patients with serious eye injuries such as lid lacerations and provide nursing services for patients in emergency rooms as well as take care of their personal hygiene. Training should be arranged and programmed for nurses to enable them manage trauma cases. Additionally, if they have patients with a serious loss of vision, they could evaluate the situation, set the differential diagnosis, manage the acute condition, and refer those patients to a required practitioner as well as consult or even educate the general practitioners since they may require training in special circumstances. In addition, ophthalmic nurses should be able to establish a firm understanding about the ophthalmologic techniques used in screening some of the most common cases of eye problems. Furthermore, nurses should be able to establish a healthy nursing relationship with their patients using their communication skills and nursing ethics to help improve follow-up procedures and create patient trust (3-10).

## Preventive ophthalmology

There are considerable advantages to prevention versus treatment. Taking preventive measures saves money and time for both patients and health-care providers. In today’s health and medical care systems, special attention has been given to screen and prevent disease. In developing countries, with regard to preventive care of ophthalmological health, health workers have become knowledgeable enough to pre-diagnose and handle ocular or systematic diseases. These workers have also assisted in emergency conditions when delays in medical services might lead to complete loss of vision or impairment. Trained nurses carry out the training of these individuals to assist them in learning about many systemic and visual disorders. According to statistics, there are around 285 million people around the world that suffer from vision impairment, 39 million of this population are completely blind and the remaining have some degree of vision impairment from moderate to severe. More than 80% of all these cases could have been prevented or treated if action had been taken at the onset of these conditions (11-14).

## Disease prevention through trained ophthalmic nurses

Untreated or prolonged diabetes mellitus is the main cause of diabetic retinopathy and in many cases that pathology will lead to complete visual loss. There are also many patients with diabetes mellitus type 2, who have no knowledge of being diabetic due to the asymptomatic nature of early stages of the disease. Once diagnosed, nurses could educate these types of people on proper diet for their problem so that they could adopt a lifestyle that would minimize health risks. In addition, nurses can assist them in taking preventive measures to save their eyes from damage and impairment. These nurses’ assistance in teaching diabetic patients to receive regular eye examinations and other health measures could help to prevent the onset of diabetic retinopathy and its subsequent complications. The sub-specialized nurses could fill the gap that usually exists between physicians and less-trained personnel and help make tremendous improvements in the health-care system and treatment results.

 Although some conditions such as exotropia and esotropia are not preventable, their early diagnosis could help to avoid amblyopia caused by strabismus. This is a common condition when one of the eyes has a high refractive error and the patient gradually relies on only his healthy eye. It is sometimes difficult for parents to detect the problem, and if undiagnosed, the child could subsequently suffer from amblyopia. By avoiding delays in services and starting treatment early, providing necessary medical treatment for children older than six could dramatically improve the chances for them to regain their vision. However, by taking a vision-screening test, children who require ophthalmic assistance would receive appropriate care. By communicating with ophthalmic nurses, mothers could learn how to detect vision problems in their children. A nurse could then handle the case by referring the patient to an ophthalmologist and prevent the future risk of blindness or loss of vision.

 In cases such as cataracts, the early diagnosis and detection of the problem in its early phase could lead to better visual outcomes and might prevent any additional defects from occurring. There are many risk factors in a growing cataract; however, the most powerful factor is age. Diabetes, unhealthy diet, unprotected exposure to sun, smoking and dehydration, are risk factors that can accelerate cataract development. Therefore, ophthalmic nurses could teach people at risk how to control these risk factors and significantly reduce the incidence of complications and disease. 

 Another ophthalmic problem is glaucoma that is classified as optic neuropathy. The most common cases of glaucoma are open-angle glaucoma, which is difficult to detect in its early stages because the causes leading to this condition are not well known. However, once primary open-angle glaucoma or other types of this disease are diagnosed, it becomes possible to control or prevent further complications through medical treatments or surgical operations. Diagnosis and treatment in the early stages should be considered as successful measures of patient care. In this circumstance, nurses are able to identify the signs and symptoms that reveal glaucoma including fast, progressive visual loss. For instance, the complications that come along with glaucoma medications have been recognized. Secondary glaucoma could be the result of taking steroids for a long time, so patients who take steroids should be informed of this possible long-term complication. There should be arrangements for routine glaucoma screenings in all who have certain risk factors. Ophthalmologic nurses could perform screenings by testing visual fields. Nurses could be trained to conduct more complicated tests such as intraocular pressure measurements in order to detect those at risk and diagnose the problem early. Training to nurses about management of ocular trauma like injuries caused in sports and the available guidelines would be mandatory. As stated before, there have been numerous cases of nurses further developing their knowledge in order to become good assistants to physicians. Many ophthalmologic nurses have been able to pre-diagnose glaucoma and other relevant diseases. This group of ophthalmic nurses usually refers more cases of acute ophthalmology problems to specialists for more overall and expert assessments of cases (1-4).

## CONCLUSION

To sum up, ophthalmic nurses have the potential to significantly contribute in ophthalmology and the visual sciences. As mentioned, there are many ocular problems that could be diagnosed and detected by well-trained ophthalmologic nurses. These trained nurses may help to reduce the rate of on-table cancelled surgical operations that had been due to lack of attention to health problems in pre-operative assessments such as uncontrolled diabetes or hypertension. Ophthalmic nurses could perform some pre-evaluation for patients who are candidates for surgeries in order to assist the healthcare systems post-surgical satisfaction rate. In addition, their services would be beneficial in helping to accelerate how quickly patients are discharged from medical facilities, which would result in less drain on financial resources in medical centers. Ophthalmic nurses are also a critical element in health-care system because they can deliver up-to-date knowledge between health-caregivers, such as general physicians. Ophthalmic nurses are able to connect patients who suffer from blindness or loss of vision to national organizations that can further assist them. Nurses are greatly able to assist in providing referrals to patients who need subspecialty services and subsequently, this will contribute to saving health-care expenditures since patients would receive proper management. In summary, ophthalmic nurses could play a significant role in the process of teaching people, providing proper diagnoses, administration and even management of many cases of medical conditions.
